# Adaptive and maladaptive emotions in patients with cancer: A network perspective

**DOI:** 10.1371/journal.pone.0341290

**Published:** 2026-10-08

**Authors:** G. Elise Doppenberg-Smit, Adriaan W. Hoogendoorn, Annemarie M.J. Braamse, Jan Willem B. de Groot, Rutger Koornstra, Evelien J.M. Kuip, Rune A.W. van de Wetering, Lia van Zuylen, Henk M.W. Verheul, Joost Dekker, Myra E. van Linde, Aartjan T.F. Beekman, Femke Lamers

**Affiliations:** 1 Department of Psychiatry, Amsterdam University Medical Center, Vrije Universiteit Amsterdam, Amsterdam, The Netherlands; 2 Amsterdam Public Health, Mental Health program, Amsterdam, The Netherlands; 3 Cancer Centre Amsterdam, Cancer Treatment and Quality of Life, Amsterdam, The Netherlands; 4 Department of Medical Psychology, Amsterdam University Medical Center, University of Amsterdam, Amsterdam, The Netherlands; 5 Isala, Oncology Center, Zwolle, The Netherlands; 6 Department of Medical Oncology, Rijnstate Hospital, Arnhem, The Netherlands; 7 Department of Medical Oncology, Radboud Medical Center, Nijmegen, The Netherlands; 8 Department of Anesthesiology, Pain and Palliative Care, Radboud Medical Center, Nijmegen, The Netherlands; 9 Department of Medical Oncology, Slingeland Ziekenhuis, Doetinchem, The Netherlands; 10 Department of Medical Oncology, Amsterdam University Medical Center, Vrije Universiteit Amsterdam, Amsterdam, The Netherlands; 11 Erasmus Medical Center, Department of Medical Oncology, Rotterdam, The Netherlands; PLOS: Public Library of Science, UNITED KINGDOM OF GREAT BRITAIN AND NORTHERN IRELAND

## Abstract

**Background:**

Receiving a cancer diagnosis frequently evokes a wide range of emotions. Emotions may help to cope with cancer: these are adaptive emotions. When emotions become disproportionate to the situation or interfere with functioning, emotions are maladaptive. According to the network theory of mental disorders, symptoms are reciprocally associated with each other; if there are strong connections between symptoms, the symptom network remains activated. A strongly connected network indicates maladaptive emotions.

**Objective:**

To examine whether patients with cancer with maladaptive emotions are characterized by stronger associations among emotional (and among emotional and cancer-treatment-related) symptoms as compared to patients with adaptive emotions.

**Methods:**

Patients with cancer (n = 132) completed Ecological Momentary Assessments for 14 days, 5 times a day, 3–7 months after start of systemic treatment. Patients were categorized as having adaptive or maladaptive emotions, based on a reference standard (psychiatric diagnostic assessment or self-reported subjective need for professional mental healthcare). Multilevel Vector Autoregression models were built in R (mlVAR package), and associations among symptoms in networks were compared (mnet package).

**Results:**

Patients with maladaptive emotions did not show stronger associations among symptoms than patients with adaptive emotions. This result of no statistically significant differences was consistently found for both emotional and cancer-treatment-related symptoms, both reference standards (psychiatric diagnostic assessment and subjective need for professional mental health care), and both contemporaneous and temporal networks (maximal edge differences ≤0.14).

**Conclusion:**

Contrary to our hypothesis, associations among emotional symptoms and of emotional and cancer-treatment-related symptoms were not stronger in patients with maladaptive emotions, as compared to patients with adaptive emotions. This raises doubts about the validity of network theory as an explanation for maladaptive emotions in patients with cancer. Since network strength did not differentiate between adaptive and maladaptive emotions, clinical assessment should continue to be guided by symptom intensity, patient functioning, and the expressed need for mental health care.

## Introduction

The occurrence of cancer constitutes a major stressor, leading to a wide range of emotions. In the field of oncology, the concept of ‘distress’ is used to refer to emotions associated with cancer. Distress refers to “a multifactorial unpleasant emotional experience of a psychological (i.e., cognitive, behavioral, emotional), social, and/or spiritual nature that may interfere with the ability to cope effectively with cancer, its physical symptoms, or its treatment” [1]. Measurement instruments (e.g., Distress Thermometer) are available to identify patients with clinically relevant distress [1]. The prevalence of clinically significant distress has been reported as being 35% to 52% [2,3].

Emotions in relation to a life-changing event such as a cancer diagnosis are adaptive: they help to cope with the disease and treatment [4,5]. For a minority of patients these emotions become disproportionate to the situation and cause impairment in daily life functioning, these emotions are considered maladaptive [4,5]. Patients with maladaptive emotions may need professional mental healthcare to cope with these emotions. Therefore, it is important to distinguish between patients with adaptive and maladaptive emotions. Current screening methods such as the Distress Thermometer only assess the intensity and not the nature of emotions: adaptive or maladaptive [5].

We postulate that network theory can be used to distinguish between patients with adaptive and maladaptive emotions [4]. According to the network theory of mental disorders, symptoms form networks, where symptoms may be more or less strongly associated with each other [6]*.* With regard to emotions in patients with cancer, network theory predicts that if strongly associated, emotional symptoms continue to be activated and may interfere with patients’ functioning; that is, these emotions become maladaptive. If not strongly associated, emotional symptoms subside and do not interfere with functioning; these emotions are adaptive and support patients’ functioning. Further, cancer-treatment-related symptoms such as fatigue or dyspnea can be present within the symptom network. Cancer-treatment-related symptoms can become strongly connected to emotional symptoms, which may contribute to emotions being maladaptive.

Evidence supporting the core assumption of network theory that symptom networks are stronger in patients with psychopathology (or maladaptive emotions) is mixed, with studies finding and not finding support for this assumption [7,8]. Prior studies using network analysis in oncology samples have typically used a cross-sectional design, and only a few studies tested differences between subgroups, using symptom severity as a criterion (e.g., [9–11]). No studies have tested whether the strength of networks differs between patients with adaptive and maladaptive emotions, using longitudinal data. To fill this research gap, the aim of the present study was to compare the association among symptoms in patients with adaptive and maladaptive emotions based on intensive longitudinal data. Based on the network theory of mental disorders, we hypothesized that a) patients with maladaptive emotions are characterized by a stronger association among emotional symptoms, compared to patients with adaptive emotions, and b) patients with maladaptive emotions are characterized by a stronger association between emotional and cancer-treatment-related symptoms, compared to patients with adaptive emotions.

## Methods

### Design

The study was part of a broader observational study on adaptive and maladaptive emotions in patients with cancer (see for previous results on this study population [12]). The study was conducted among patients with a solid malignancy at two academic and four non-academic hospitals in the Netherlands. Emotional symptoms (positive and negative emotions) and cancer-treatment-related symptoms were assessed with Ecological Momentary Assessment (EMA). Patients were followed for two weeks at 3–7 months after the start of chemo- or immunotherapy. To differentiate between patients with adaptive versus maladaptive emotions a reference standard was used: (a) psychiatric diagnostic assessment, or (b) the patient’s subjective need for professional mental healthcare. This design was repeated in its entirety three months after the first assessment (i.e., 6–10 months after the start of chemo- or immunotherapy). Data collection took place between August 10^th^, 2021 and May 6^th^, 2024. The study was registered on clinicaltrials.gov (NCT05062447) and was approved by the Medical Ethics Committee of Amsterdam UMC (2020.099 - NL72824.029.20). All participants provided written informed consent.

### Sample

Patients were eligible if they received a diagnosis of a malignant solid tumor (non-metastasized or metastasized), started with (neo)adjuvant (treatment for non-metastasized disease) or first line (treatment for metastasized disease) chemo- or immunotherapy 3–7 months prior to enrollment, and had a life expectancy of more than 3 months. Exclusion criteria were: age < 18; no sufficient understanding of the Dutch language; presence of psychopathology other than mood or anxiety disorder (e.g., lifetime history of bipolar disorder, schizophrenia, major depressive disorder with psychotic features, schizoaffective disorders); severe cognitive impairments (e.g., dementia); (indication of) brain tumor or brain metastases; a history of severe drug or alcohol abuse within past 6 months; not competent in the use of a mobile phone; and providing no informed consent. Patients were approached by their treating oncologist or nurse (specialist) to participate in this study.

### Screening

We aimed to include 80 patients with adaptive and 80 with maladaptive emotions. Because patients with maladaptive emotions are a minority, after 80% of patients with adaptive emotions were included we implemented a screening procedure (see S1 Appendix).

### Procedure

For consenting patients, the assessment started with an appointment with the researcher in the hospital or through video call. The participants first received training in the EMA, which is explained further below. After that, an interview as part of the psychiatric diagnostic assessment was conducted. The training and the interview were conducted by a trained researcher (ED) or by a trained research assistant. After the appointment with the researcher, the EMA started and participants were asked to fill out additional online questionnaires. After three months, the entire assessment procedure was repeated. See Fig 1 for an overview of the assessments.

**Fig 1:**
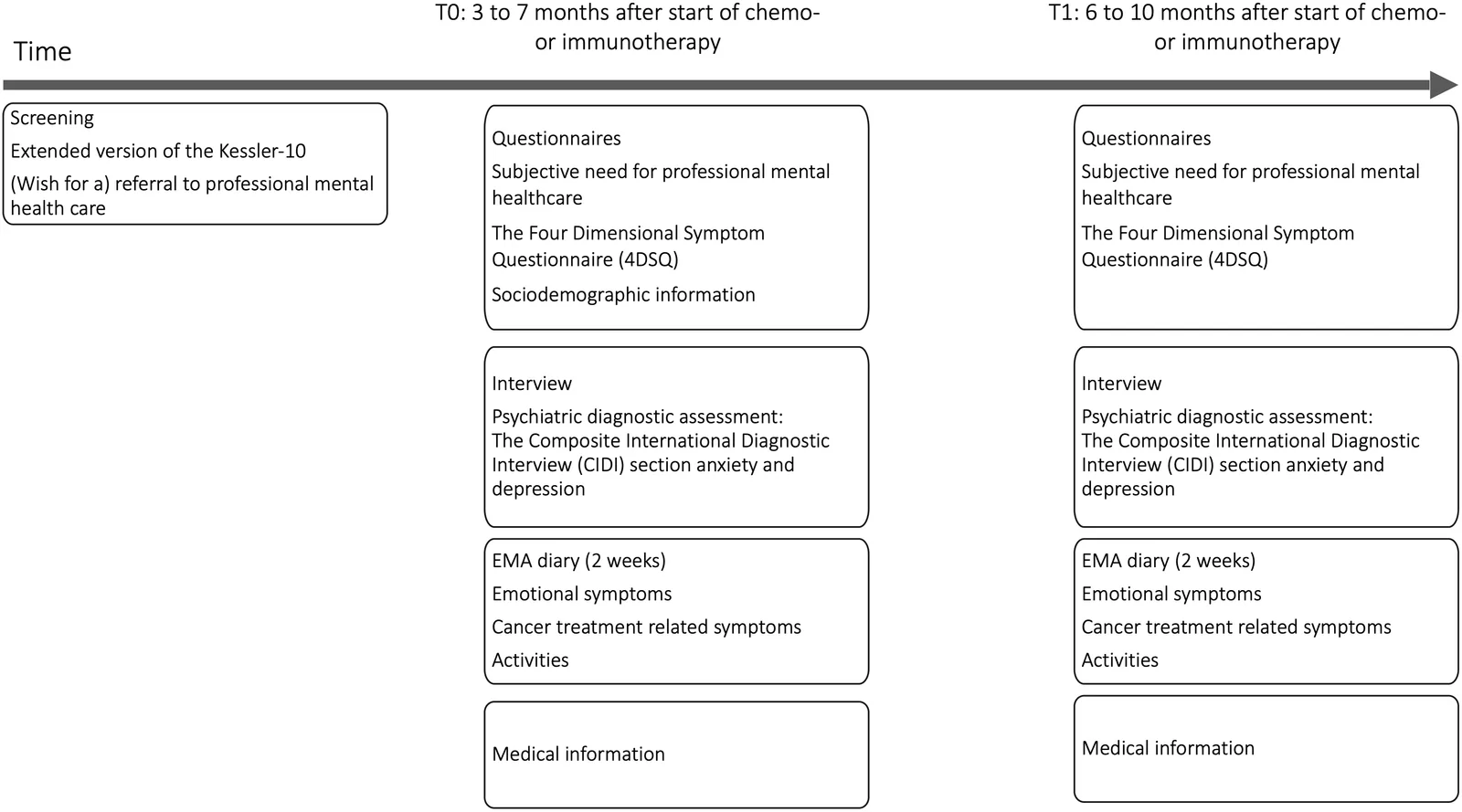
Overview of assessments.

### Measures

Sociodemographic information was self-reported using an online questionnaire developed for this study. Medical information was retrieved from the medical record. This entailed information about the cancer diagnosis, metastasis (yes/no), timing of diagnosis and first treatment, comorbidities and adverse events grade 3 or higher, according to the Common Terminology Criteria of Adverse Events 5.0 [13].

### Emotional and cancer-treatment-related symptoms

Momentary emotional symptoms and cancer-treatment-related symptoms were assessed via mobile phone during 14 consecutive days, 5 times per day using EMA. A text message was send out with a personalized link to the questionnaire using the secured server system RoQua [14]. A fixed assessment protocol was used to obtain time-series data with equidistant time points [15]. Participants were instructed to complete the questionnaire as soon as possible after receiving the prompt. In order to give patients enough time to respond, the questionnaire was available for 60 minutes after the prompt [15]. A reminder to fill in the questionnaire was sent after 30 minutes. The full EMA protocol consisted of 15 emotional and 6 cancer-treatment-related symptoms, followed by 5 questions on activities since the last assessment, and where and with whom they were at the moment of the assessment. For current analyses, only the emotional and cancer-related symptoms were used. Emotions with a negative valence included feeling irritated, upset, listless/apathetic, down, nervous, bored, anxious, and worried. Emotions with a positive valence included feeling satisfied, relaxed, cheerful, energetic, enthusiastic and calm. Cancer-treatment-related symptoms included feeling tired, difficulty concentrating, nauseous, lack of appetite, pain and shortness of breath. Patients rated emotions and symptoms on a 7-point scale (1 = not at all; 7 = very much). The emotions were derived from the Netherlands Study of Depression and Anxiety (NESDA) EMA diary-protocol [16], and have been extensively used in previous studies [17–20]*.* The cancer related symptoms were derived from the European Organisation for Research and Treatment of Cancer item bank [21]. See S2 Appendix for the full EMA protocol (translated from Dutch) as used in this study.

### Reference standards

Patients were categorized as experiencing either adaptive or maladaptive emotions, using one of two reference standards. (a) *Psychiatric diagnostic assessment.* The Composite International Diagnostic Interview version 2.1 (CIDI) section Anxiety and Depression is a full-structured diagnostic interview that is used worldwide to assess current (one month recency) and lifetime presence of a depressive or anxiety disorder according to the DSM-IV criteria [22]. The interrater reliability for the CIDI interviews is high [22]. In the present study, the interviews were conducted by a well-trained researcher (ED) or a research assistant, who were supervised by a psychiatrist (ATFB). The Four Dimensional Symptom Questionnaire (4DSQ) was used to assess adjustment disorder [23], using >20 on Distress and without current anxiety or depression on the CIDI to categorize patients as having an adjustment disorder. A one-month recency diagnosis of anxiety or depressive disorder on the CIDI or a score above the cut-off on the Distress scale of the 4DSQ were considered indicators of maladaptive emotions. (b) *Subjective need for professional mental health care.* Patient’s subjective need for professional mental healthcare was assessed with the Problem List [24]. The Problem List is a self-reported questionnaire that maps problems across several domains [24]. The emotional domain was used to evaluate subjective need for professional mental healthcare. Patients indicated whether in the last three months they experienced a problem in this domain, using a five-point scale (ranging from 1 ‘no problem at all’ to 5 ‘very much a problem’), as was done in previous studies [25,26]. When participants reported at least one emotional problem, they were asked whether they (i) received a referral for the problem, (ii) did not receive a referral, but would have preferred a referral for the problem, (iii) did not want or received a referral for the problem, but would be willing to consider a referral in the future, or (iv) did not want or received a referral, neither in the present nor the future. Maladaptive emotions were rated present if the patient had received a referral or would have preferred to have received a referral for emotional problems on the Problem List [25,26].

### Statistical analyses

All analyses were conducted in R version 4.3.2 [27]. Before networks were formed, the assumptions of sufficient stationarity and variability were checked. Because not all variables showed sufficient variability, the results were cross-validated (See S3 Appendix for assumptions and cross-validation). Patients with >50% missings were excluded from the analysis. The Kalman smoothing was used for imputing remaining missing items [28–30], using the R package ‘ImputeTS’ version 3.2’ [31].

### Building networks

Our data had a hierarchical structure: measurements within days within patients. To form networks, we therefore conducted a three level Vector AutoRegression analysis using the package ‘mlVAR’ version 0.5.2 [32]. Separate networks were formed for patients with adaptive and maladaptive emotions, based on one of the two reference standards. The first set of networks consisted of emotions with a negative valence (hypothesis a), the second set consisted of emotions with a negative valence and cancer-treatment-related symptoms (hypothesis b), and in an exploratory analysis, the third set of networks consisted of emotions with a positive valence. Both contemporaneous and temporal networks were formed. Contemporaneous networks are undirected networks that estimate partial correlations (edges) between selected variables (nodes) assessed within the same time window [33]. Partial correlations are associations between two nodes while controlling for all other nodes. Temporal networks have partial directed correlations, where variables at a given timepoint are predicted by the same variable at an earlier timepoint (autoregressive association) as well as by all other variables at earlier timepoints (cross-lagged association) [34]. To improve the robustness as well as interpretability of the results, correlations <0.10 were considered negligible and were removed from the networks. Because of the difference in sample size of the adaptive and the maladaptive groups, edges were removed based on strength of the association (correlation <0.10), instead of the usual statistical significance. The correlation matrices including all associations in the networks (including correlations <0.10) can be found in S1-S3 Matrices.

### Network comparison

To compare the strength of the associations in the networks of patients with adaptive or maladaptive emotions the ‘mnet’ package (version 0.1.4) in R was used [35]. This package calculates the difference between all edges in two networks (edge network 1 minus that same edge in network 2) and tests the statistical significance (p < 0.05) of the difference [36]. Additionally, to compare the one level networks of patients with adaptive and maladaptive emotions, the Network Comparison Test was used [37].

### One-level networks and sensitivity analyses

As a robustness check, one level networks were formed, based on the averaged item scores of the 70 EMA per patient, separately for patients with adaptive and maladaptive emotions. As a sensitivity analysis, networks were derived separately for patients with metastatic and non-metastatic disease. Additionally, the results of the first measurement were compared to results of the second measurement (3–7 months and 6–10 months after the start of treatment with chemo- or immunotherapy, respectively).

### Power calculation

When the study was set up, no power calculation was available yet for applying the network approach to EMA data. We aimed to recruit 160 patients. For each patient, 70 measurements, over 14 days with 5 repeated measurements were intended. These numbers were similar to the corresponding numbers in a review [38].

## Results

### Sample description

Of the 134 patients included in the study, two patients dropped out early and did not complete ≥50% of the EMA. Hence, for the current analyses we used data from 132 patients. See Table 1 for patient characteristics. Patients were between 22 and 86 years old and the majority was diagnosed with gastro-intestinal cancer, breast cancer, or melanoma. Patients with non-metastatic disease comprised 56% of the sample. Based on the psychiatric diagnostic assessment, 23.5% experienced maladaptive emotions (versus 76.5% adaptive emotions). Based on the subjective need for professional mental health care, 26.5% experienced maladaptive emotions (versus 73.5% adaptive emotions). Roughly half of the sample with maladaptive emotions according to one reference standard was also grouped as having maladaptive emotions according to the other reference standard. The average score on negative emotions ranged from 1.28–3.04 (see S1 Table). All negative emotions were more severe in the group of patients with maladaptive emotions, apart from Bored and Listless with the subjective need for professional mental health care reference standard. For positive emotions, all six emotions were significantly lower for patients with maladaptive emotions with the psychiatric diagnostic assessment reference standard, while only one positive emotion (Cheerful) was significantly lower for patients with maladaptive emotions with the subjective need for professional mental health care reference standard. None of the cancer-treatment-related symptoms differed significantly between patients with adaptive versus maladaptive emotions, apart from Lack of Concentration with the psychiatric diagnostic assessment reference standard (see S1 Table). None of the negative emotional and cancer-treatment-related symptoms differed between patients with metastatic versus non-metastatic disease, apart from Shortness of Breath: patients with metastatic disease had a significantly higher score indicating more shortness of breath. Regarding positive emotions, patients with metastatic disease showed a higher score on Cheerful, Energetic and Enthusiastic (see S2 Table).

**Table 1:** Patient characteristics (N = 132).

	n (%)/ *mean (SD)*
Age (range 22–86)	*57.7 (12.9)*
Gender	
Female	77 (58.3%)
Male	55 (41.7%)
Diagnosis	
Gastro-intestinal cancer	34 (25.8%)
Breast cancer	33 (25.0%)
Melanoma	31 (23.5%)
Urogenital cancer	12 (9.1%)
Gynecological cancer	8 (6.1%)
Sarcoma	7 (5.3%)
Head/Neck cancer	5 (3.8%)
Other	2 (1.5%)
Metastatic disease	
Yes	58 (43.9%)
No	74 (56.1%)
Psychological help in past year	
No	100 (75.8%)
Yes	32 (24.2%)
Psychiatric diagnostic interview	
Adaptive emotions	101 (76.5%)
Maladaptive emotions	31 (23.5%)
Subjective need for help	
Adaptive emotions	97 (73.5%)
Emotions	35 (26.5%)

Information from this table has already been published in [12]. Despite the same total number of participants, there are slight differences between the two tables due to study-specific selection criteria.

### EMA measurements

The targeted number of EMA measurements was 9240 (132 patients x 14 days x 5 EMA measurements per day). The attained number of EMA measurements was 8603, implying a 93.1% response rate which indicates that the amount of missing information at this level was limited. Regarding the within-person variability in the EMA measurements, we studied the variability of fourteen items of negative emotions within 132 patients. We found that 50.9% of the (14x132) patient-by-item combinations showed low variation (meaning that the within-person SD was less than 10% of the range of the item scale, i.e., SD < 0.6). Furthermore, almost half of the patient-by-item combinations did not show any variation at all (i.e., SD = 0). Still, the models did not show any problem in convergence and provided parameter estimates with standard errors for the edges in the contemporaneous and temporal network models. An anonymized and minimal dataset (only variables of the first measurement needed to build the networks) and excluding patients who did not consent for their data to be shared, is available on https://osf.io/mr9wt/. Additionally, cross validation of the network analyses with negative emotional symptoms showed that on average 75% of the edges in the contemporaneous networks and 79% of the edges in the temporal networks were also retrieved in >800 of the 1000 iterations of the analyses (see S4 Appendix). This demonstrated the robustness of the generated networks.

### Networks of negative emotions

#### Contemporaneous networks.

The edges in the contemporaneous networks were rather weak, the largest partial correlation was 0.35. The strongest edges were between Down and Listless and between Nervous and Anxious in all four networks (i.e., the adaptive and maladaptive networks for both reference standards; see Fig 2). Down was the symptom with the most edges to other emotional symptoms, and Bored was the symptom with the lowest number of edges within all four networks.

**Fig 2:**
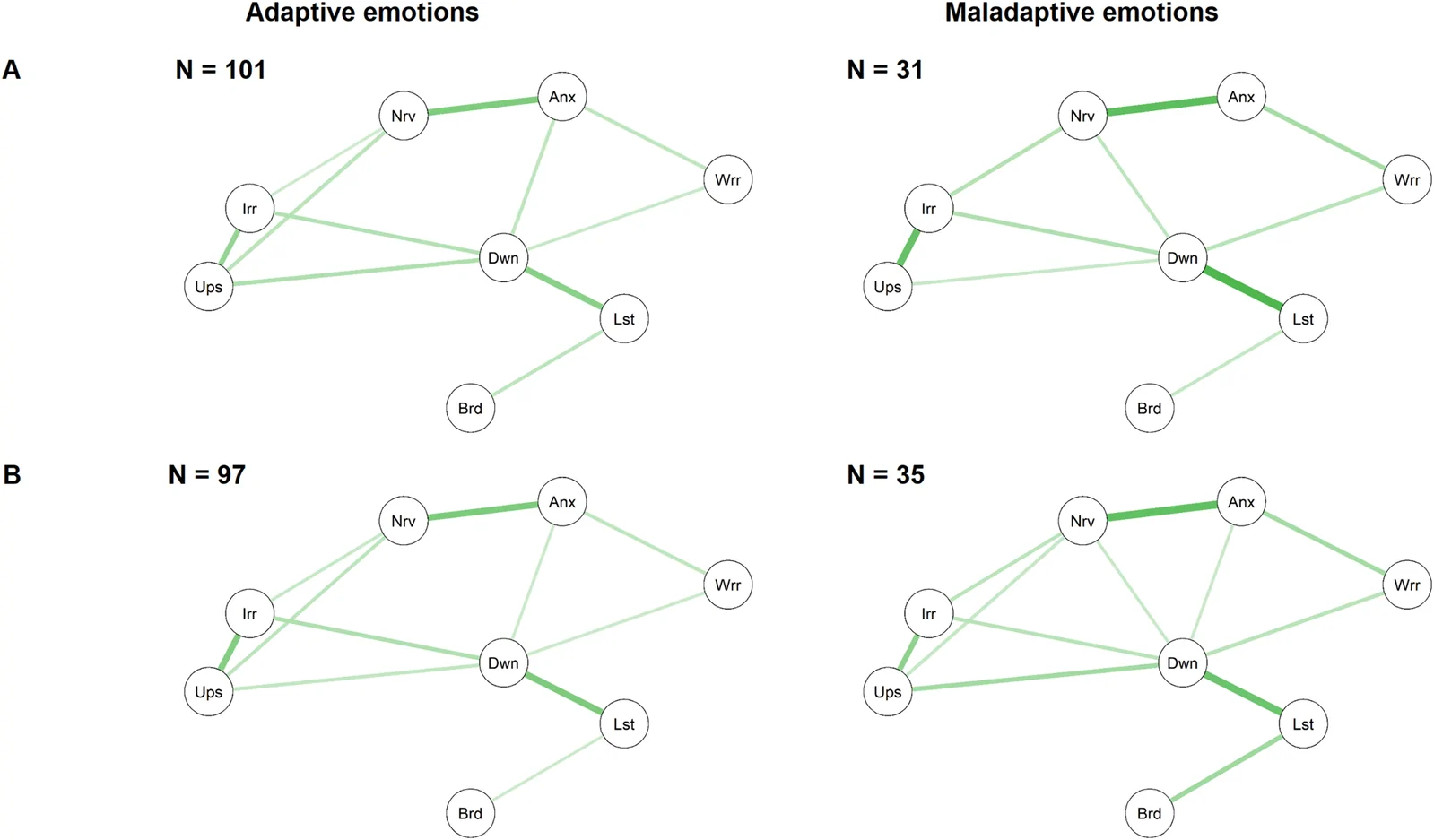
Contemporaneous networks with negative emotions. Contemporaneous networks of patients with adaptive and maladaptive according to (A) the psychiatric diagnostic assessment reference standard and (B) the subjective need for professional mental health care reference standard. Thicker edges show a greater connection between two symptoms. All edges are positive.“Anx” = “Anxious”, “Brd” = “Bored”, “Dwn” = “Down”, “Irr” = “Irritated”, “Lst” = “Listless”, “Nrv” = “Nervous”, “Ups” = “Upset”, “Wrr” = “Worried”.

The comparison of the networks showed no statistically significant differences between the edges of the adaptive and maladaptive networks, with two exceptions for the psychiatric diagnostic assessment networks (one stronger in the adaptive network and one stronger in the maladaptive network), and one exception in the networks based on the subjective need for professional mental healthcare reference standard (stronger for the adaptive network; S1 Fig). All differences were small (≤0.12; see S1-S3 Matrices for all correlation matrices).

#### Temporal networks.

The temporal networks had few edges larger than 0.10 (Fig 3). The comparison of the networks showed no statistically significant differences between the edges of the adaptive and maladaptive networks, apart from Bored (stronger in patients with maladaptive emotions with the psychiatric diagnostic assessment reference standard and stronger in patients with adaptive emotions with the subjective need for professional mental healthcare reference standard). Cross-lagged associations were not statistically significantly different between patients with adaptive or maladaptive emotions with the following exceptions. For the networks using the psychiatric diagnostic assessment as reference standard, two cross-lagged associations were stronger in the network from patients with maladaptive emotions, and two cross-lagged associations were stronger in the network of patients with adaptive emotions. For the networks using the subjective need for professional mental healthcare standard, three cross-lagged associations were significantly stronger in the network from patients with adaptive emotions (S2 Fig). All differences were small (≤0.11).

**Fig 3:**
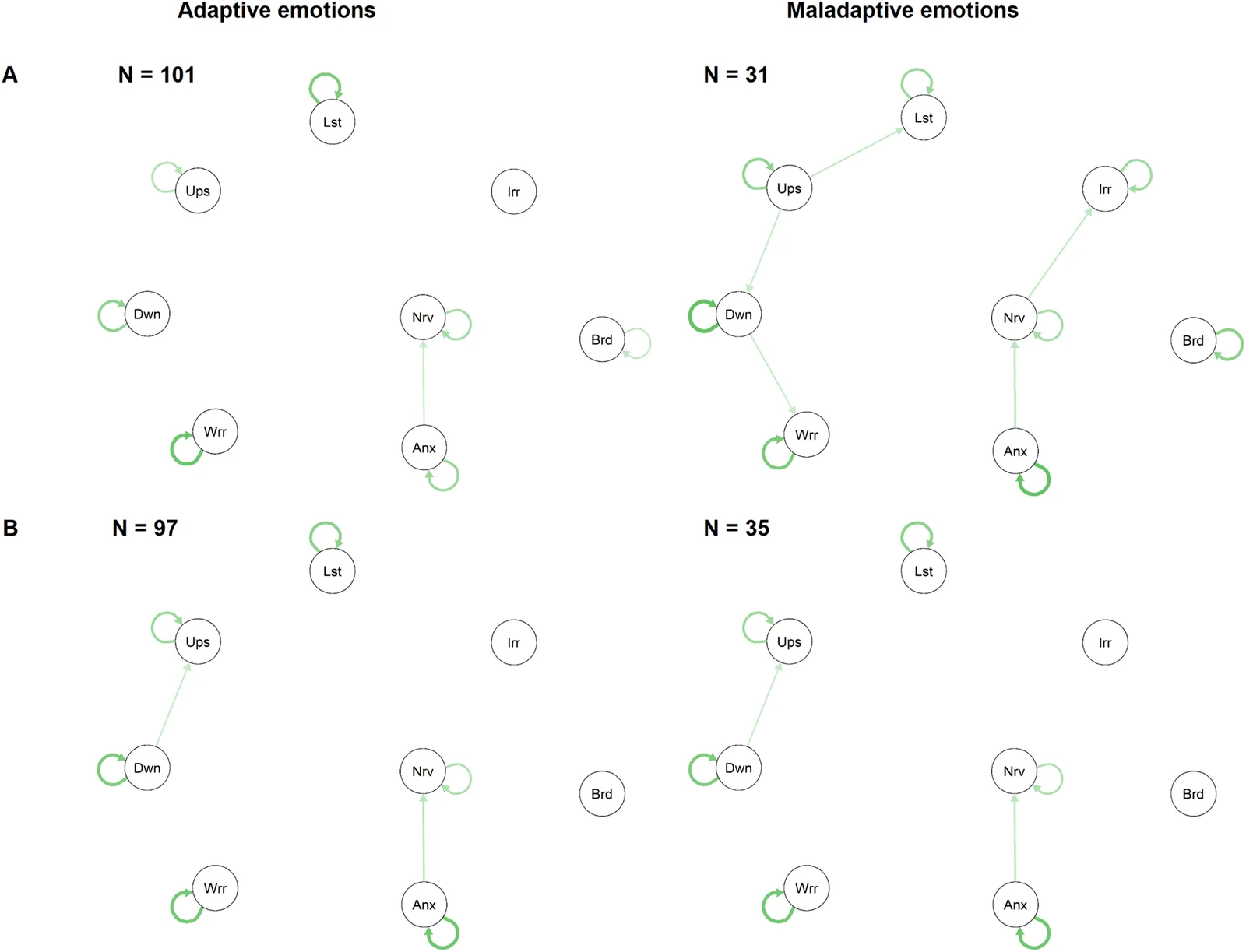
Temporal networks of negative emotional symptoms. Temporal networks of patients with adaptive and maladaptive according to (A) the psychiatric diagnostic assessment reference standard and (B) the subjective need for professional mental health care. Thicker edges show a greater connection between two symptoms. All edges are positive. “Anx” = “Anxious”, “Brd” = “Bored”, “Dwn” = “Down”, “Irr” = “Irritated”, “Lst” = “Listless”, “Nrv” = “Nervous”, “Ups” = “Upset”, “Wrr” = “Worried”.

#### One-level networks.

For the one-level networks based on the within-person averaged item scores across the 70 EMA, the global strength of the adaptive networks was 3.57 and 3.79, while the global strength of the maladaptive networks was 4.59 and 3.66 for the psychiatric diagnostic assessment and subjective need for professional mental healthcare reference standard, respectively. Thus, the comparison of the networks showed no statistically significant difference between overall strength of the adaptive and maladaptive networks (see S5 Appendix). This is in line with the results derived from the three-level network analyses.

### Networks of negative emotional symptoms and cancer-treatment-related symptoms

#### Contemporaneous networks.

The edges in the contemporaneous networks were rather weak, the largest partial correlation was 0.32. The contemporaneous networks showed that the cancer-treatment-related symptoms were rather separate from the emotional symptoms (see Fig 4). The cancer-treatment-related symptoms were only connected to emotional symptoms through Tired in all networks and Lack of Concentration in one network. Tired served as bridge symptom in all four networks (Fig 4). As in the networks with emotional symptoms alone, the edges Listless-Down, Nervous-Anxious were the strongest. In addition, Concentration-Tired and Nauseous-Tired were also among the strongest in all four networks (Fig 4).

**Fig 4:**
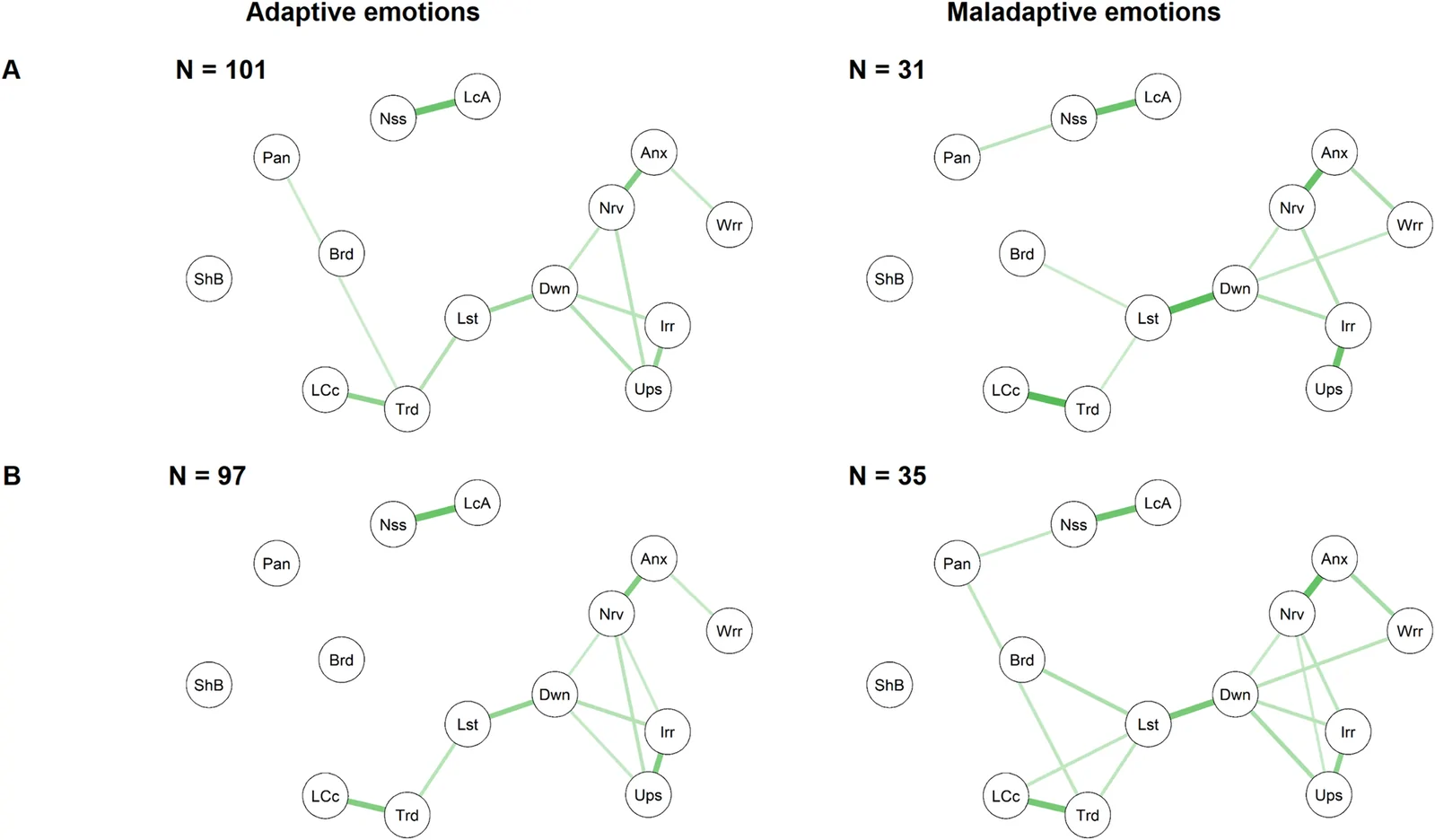
Contemporaneous networks of negative emotional and cancer-treatment-related symptoms. Contemporaneous networks of patients with adaptive and maladaptive according to (A) the psychiatric diagnostic assessment reference standard and (B) the subjective need for professional mental health care reference standard. Thicker edges show a greater connection between two symptoms. All edges are positive. Negative emotional symptoms: “Anx” = “Anxious”, “Brd” = “Bored”, “Dwn” = “Down”, “Irr” = “Irritated”,”Lst” = “Listless”, “Nrv” = “Nervous”, “Ups” = “Upset”, “Wrr” = “Worried”; cancer-treatment-related symptoms: “LCc” = “Lack of concentration”, “Nss” = “Nauseous”, “LcA” = “Lack of appetite”, “Pan” = “Pain”, “ShB” = “Shortness of breath”, “Trd” = “Tired”.

The comparison of the networks showed no statistically significant differences between the edges of the adaptive and maladaptive networks. This applied to the networks based on the psychiatric diagnostic assessment, apart from three edges (one stronger in the network of patients with adaptive emotions, and two edges stronger in the networks of patients with maladaptive emotions). For the networks based on the subjective need for professional mental healthcare, no statistically significant differences were found either, with one exception (stronger in the network of patients with adaptive emotions; see S3 Fig). All differences were small (≤0.13).

#### Temporal networks.

The four temporal networks showed a small number of cross-lagged associations. Three out of four networks had one cross-lagged association. Regarding the association between emotional symptoms and cancer-treatment-related symptoms, there was only one cross-lagged association between cancer-treatment-related symptoms and emotional symptoms across all four networks (Fig 5).

**Fig 5:**
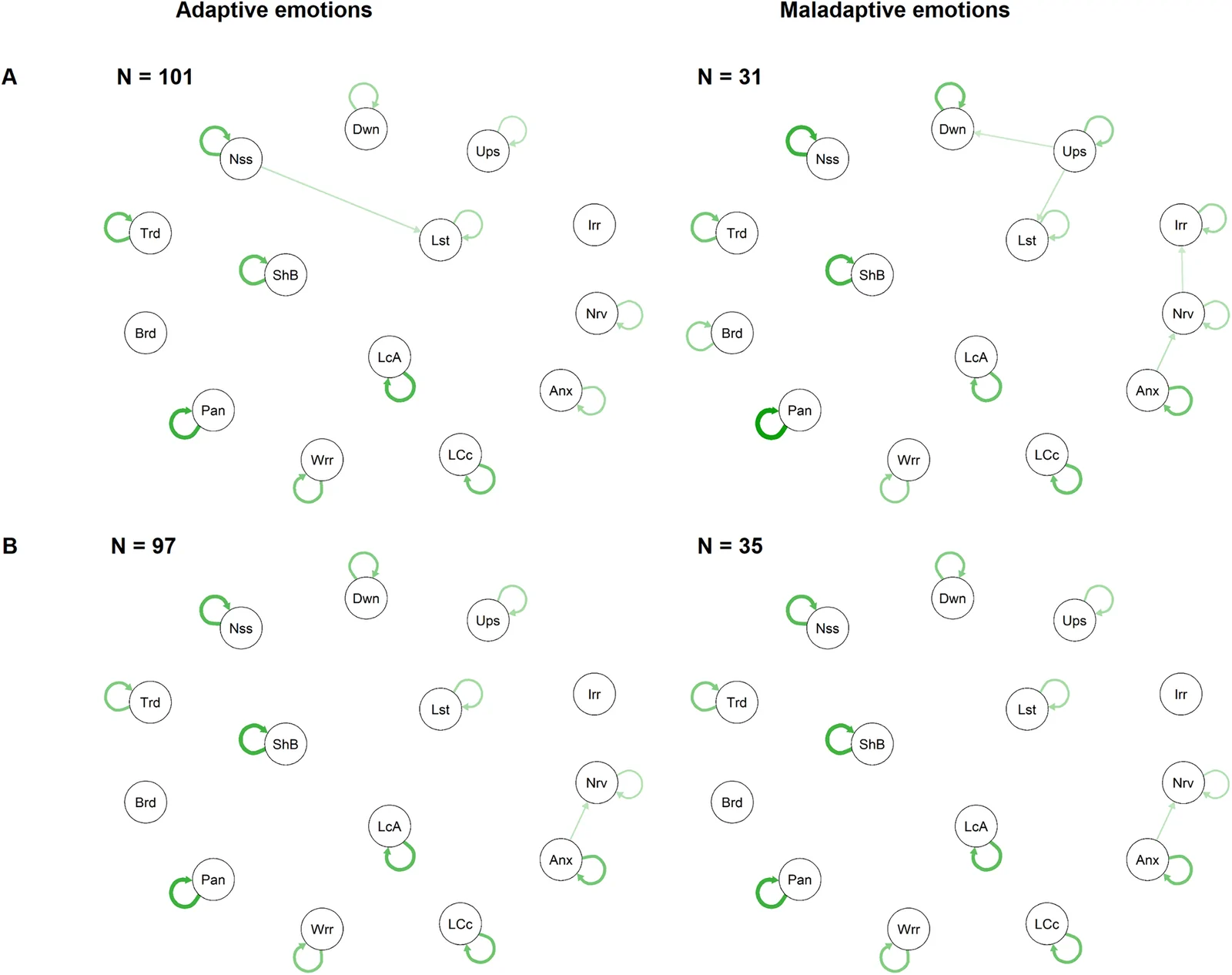
Temporal networks of negative emotional and cancer-treatment-related symptoms. Temporal networks of patients with adaptive and maladaptive according to (A) the psychiatric diagnostic assessment reference standard and (B) the subjective need for professional mental health care reference standard. Thicker edges show a greater connection between two symptoms. All edges are positive. Negative emotional symptoms: “Anx” = “Anxious”, “Brd” = “Bored”, “Dwn” = “Down”, “Irr” = “Irritated”, “Lst” = “Listless”, “Nrv” = “Nervous”, “Ups” = “Upset”, “Wrr” = “Worried”; cancer-treatment-related symptoms: “LCc” = “Lack of concentration”, “Nss” = “Nauseous”, “LcA” = “Lack of appetite”, “Pan” = “Pain”, “ShB” = “Shortness of breath”, “Trd” = “Tired”.

The comparison test showed several statistically significant differences between the adaptive and maladaptive network (11 edge-differences for the networks based on the psychiatric diagnostic assessment and seven for the subjective need for professional mental healthcare networks): 11 edges were stronger in the adaptive network, while seven edges were stronger in the maladaptive network (S4 Fig). Again, all differences were small (≤0.13).

### Sensitivity analyses

#### Second measurement.

The results of the patients who participated in the second measurement (110 out of 134 patients) were similar to the results of the first measurement, where the contemporaneous and temporal networks were not consistently stronger in the maladaptive compared to the adaptive group, for both reference standards (see S5 Fig and S6 Fig for differences).

#### Metastatic versus non-metastatic disease.

The results for patients with metastatic and non-metastatic disease were similar. For both patients with metastatic and non-metastatic disease, the differences for the contemporaneous and temporal networks were not consistently stronger in the maladaptive compared to the adaptive group, for both reference standards (see S7 Fig and S8 Fig for differences).

#### Networks of positive emotions.

The exploratory analysis with positive emotions showed that all four contemporaneous networks were alike (only one network had an edge that was not present in the other networks; Fig 6). The temporal networks were dissimilar: only the edge from Satisfied to Relaxed was present in three out of four networks (Fig 7).

**Fig 6:**
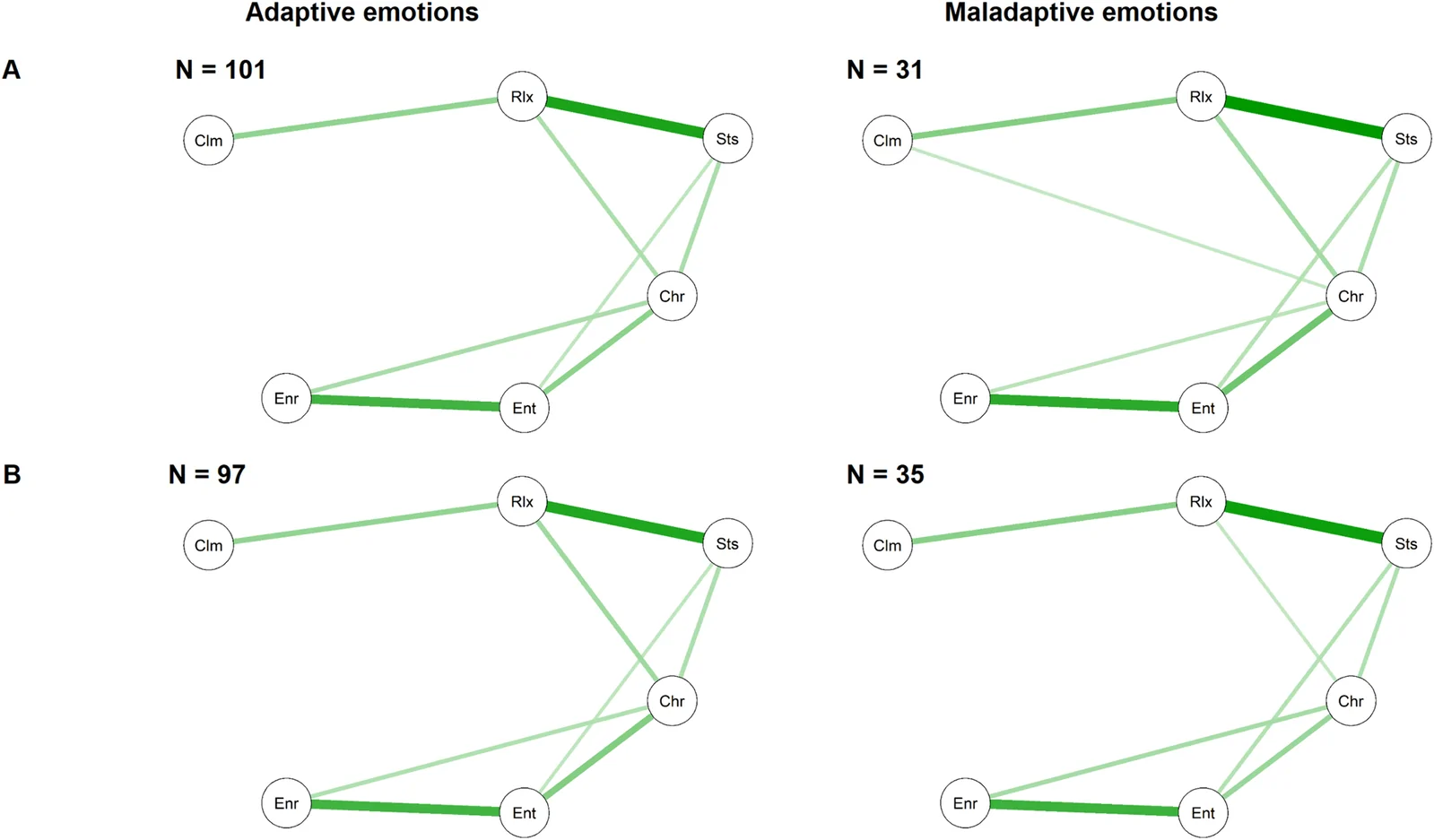
Contemporaneous networks of positive emotions. Contemporaneous networks of patients with adaptive and maladaptive according to (A) the psychiatric diagnostic assessment reference standard and (B) the subjective need for professional mental health care. Thicker edges show a greater connection between two symptoms. All edges are positive. “Chr” = “Cheerful”, “Clm” = “Calm”, “Enr” = “Energetic”, “Ent” = “Enthusiastic”, “Rlx” = “Relaxed”, “Sts” = “Satisfied”.

**Fig 7:**
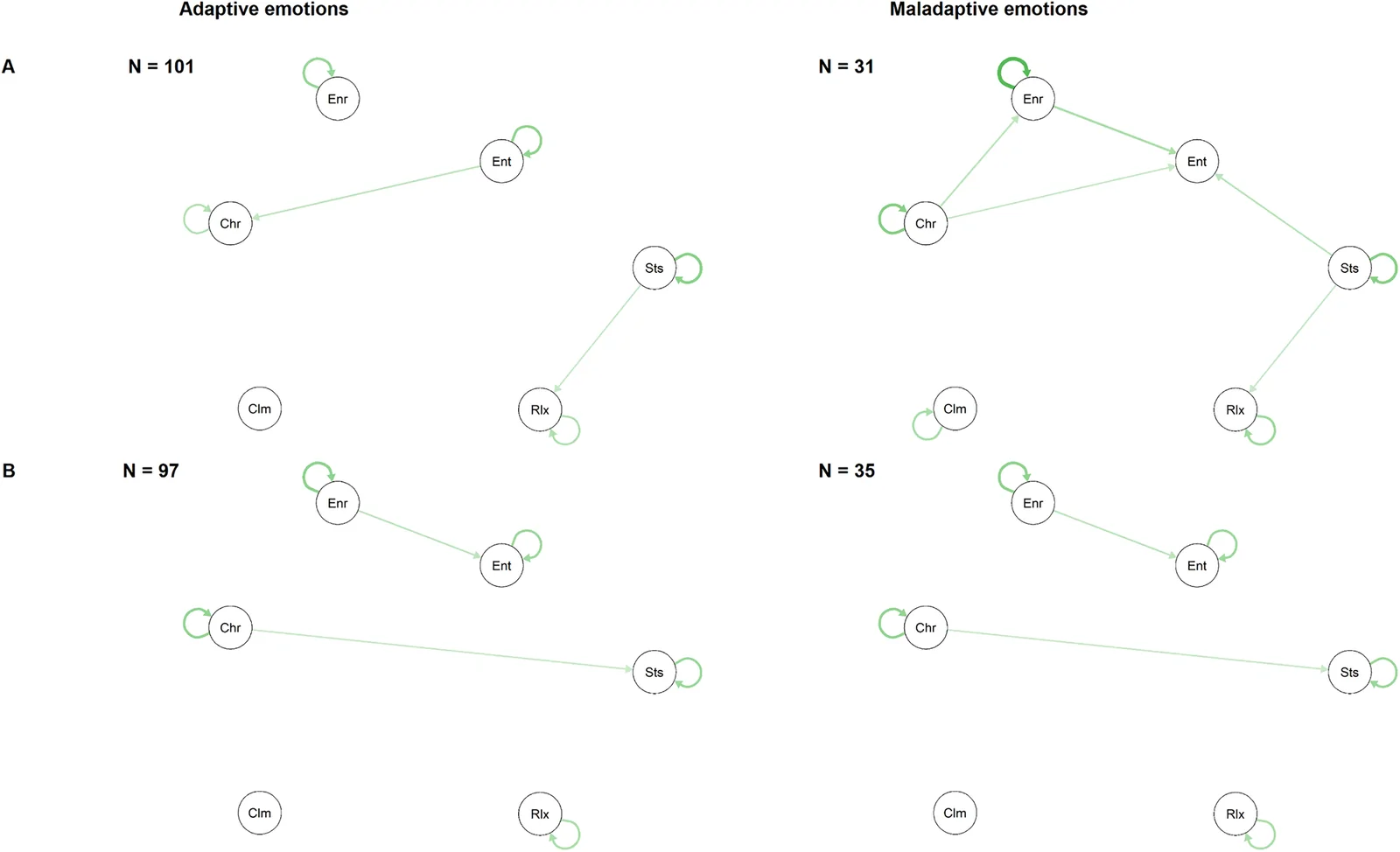
Temporal networks of positive emotions. Temporal networks of patients with adaptive and maladaptive according to (A) the psychiatric diagnostic assessment reference standard and (B) the subjective need for professional mental health care reference standard. Thicker edges show a greater connection between two symptoms. All edges are positive. “Chr” = “Cheerful”, “Clm” = “Calm”, “Enr” = “Energetic”, “Ent” = “Enthusiastic”, “Rlx” = “Relaxed”, “Sts” = “Satisfied”.

For the networks based on the subjective need for professional healthcare, no statistically significant differences were found in the strength of the edges between patients with adaptive and maladaptive emotions (S9 Fig and S10 Fig). For the networks based on the psychiatric diagnostic assessment, no statistically significant differences were found, apart from one edge in the contemporaneous network (S9 Fig) and seven statistically significant differences were found in the temporal networks, five of which involved the variable Enthusiastic (four stronger in the network of patients with adaptive emotions, one in the network for patients with maladaptive emotions; S10 Fig). All differences were small (≤0.14).

## Discussion

The present study examined emotional and cancer-treatment-related symptoms in patients with adaptive and maladaptive emotions using the network theory. Results showed that although patients with maladaptive emotions experienced more severe negative emotional symptoms, the association among negative emotional symptoms was not stronger in patients with maladaptive emotions, as compared to patients with adaptive emotions. Additionally, no differences were found between patients with adaptive and maladaptive emotions regarding the association between emotional and cancer-treatment-related symptoms. This result of no statistically significant differences was consistently found for both the contemporaneous and the temporal networks and for both reference standards (psychiatric diagnostic assessment and subjective need for professional mental healthcare). The results of the second assessment confirmed the results of the first assessment. Where, exceptionally, we did find a statistically significant difference it was sometimes in the expected direction (stronger associations in the maladaptive group) and then again the other way around (stronger associations in the adaptive group). Thus, we found no support for the hypotheses derived from the network theory. In short, the associations in all networks were generally weak and the null findings were robust across reference standards, network types, and measurement occasions. Consequently, there is limited support for the discriminative value of network strength in this setting.

To find an explanation for the lack of support, we first turn to methodological issues of our study. *Firstly,* although we screened for maladaptive emotions to create a balanced sample, the sample size of the maladaptive group was smaller than intended. Although the number of patients with adaptive emotions was higher than intended, this resulted in some decrease of power. However, the difference in the strength of the associations was very small (smaller than 0.14). If a larger and more balanced sample were to find statistically significant differences, this is unlikely to have clinical significance. *Secondly*, the variability of the symptoms was lower than expected. For a network analysis based on 70 measurements, variability is necessary to capture moment-to-moment changes. Low variability is more likely to occur in analyses using ‘real life’ data instead of simulated data, as simulated data is optimized for testing software [36,39]. It is possible that people do not shift in emotions abruptly as is desirable for creating networks. However, where the low variability is a methodological challenge, it may reflect stable affective profiles in patients with cancer. Therefore, it does not invalidate the results. In addition, the networks did converge and the cross validation supported the generated networks and our results. *Thirdly,* the reference standards dichotomized emotions as either adaptive or maladaptive (with scores above or below a certain cut off). It is possible that the adaptiveness of emotions is a continuum rather than a dichotomy. However, the interview for the psychiatric diagnostic assessment and its cut-off has been widely used to group patients as with or without a psychiatric disorder. In addition, the two reference standards showed similar results.

The fundamental assumption of network theory is that mental disorders (or maladaptive emotions) are characterized by a relatively strong association among symptoms, keeping the network activated [6]. The two most recent reviews on empirical support for this assumption found mixed evidence: while some studies supported this assumption, there were also studies that did not [7,8]. The present study also failed to support this assumption. Furthermore, the association among negative emotions was rather weak, even in patients with maladaptive emotions. One might question whether such a weak association is sufficient to keep the network activated. Thus, the lack of support for our hypothesis and the weak associations among symptoms raise doubts about the validity of network theory as an explanation for maladaptive emotions in people with cancer.

One could argue that our negative results are due to the fact that the external stressor that actives a network of symptoms was not truly vanished. The network theory assumes that after the external stressor (in this case: the experience of receiving a cancer diagnosis and related treatment) is vanished, the network returns to a stable state (adaptive emotions) or remains activated (maladaptive emotions) [6]. Based on clinical experience, we expected that 3–7 months after the start of chemo- or immunotherapy the differentiation between patients with adaptive versus maladaptive emotions would have occurred. To allow patients even more time to return to a stable state (adaptive emotions), we included a second measurement three months after the first (6–10 months after the start of chemo- or immunotherapy). However, it is a distinct possibility that patients still experienced the cancer diagnosis, related treatment and the late effects as a major stressor. Indeed, distress trajectories in patients with cancer show that, where the majority of patients experience low distress over time, others have elevated distress even long after treatment [40–42]. However, if the continued presence of the stressor is indeed the explanation for our negative findings, then this still implies that network theory is not the appropriate theoretical framework for distinguishing between maladaptive emotions that require professional mental healthcare and adaptive emotions that do not require professional care.

The network theory on mental disorders is a relatively new theory. After the first version of the theory had been formulated [6], suggestions were made to improve the theory, methodology, and statistics (e.g., [7,43–45]). In addition, more empirical research is needed, including research into the fundamental assumption that networks of patients with mental disorders are more strongly connected than networks of patients without mental disorders. In doing so, consideration may be given to operationalizing emotional adaptiveness as a continuum rather than a dichotomy and to using advanced statistical methods (e.g., dynamic structural equation modeling or Bayesian multilevel approaches) to capture symptom inertia and individual symptom heterogeneity.

This study also found weak associations between negative emotional symptoms and cancer-treatment-related symptoms. This is in line with the findings from our scoping review, showing that psychological (emotional) symptoms and somatic (cancer-treatment-related) symptoms were more strongly associated among themselves than with each other [46]. The current study showed that the emotional symptoms and cancer-treatment-related symptoms were mainly connected through Tired. This is also in line with the findings of our scoping review.

Strengths of our study include the use of Ecological Momentary Assessments that measure emotions over a longer period of time, the use of two different reference standards to group patients as having adaptive or maladaptive emotions, and the timing of the measurement: three months after start of treatment rather than at diagnosis. In addition to the abovementioned methodological challenges, the findings ought to be interpreted with the following limitation in mind: it is possible that patients with severe emotional problems were not referred to or were not willing to participate in our study. Likely, the emotions of these patients are more intense than those of the patients in our sample. This may have reduced the generalizability of our findings.

The clinical implications of these findings are that network connectivity is not a useful criterion for referring patients to professional mental health care. Screening for maladaptive emotions and the associated need for professional mental health care should continue to be guided by symptom intensity, patient functioning, and the expressed need for mental health care.

In conclusion, contrary to our hypothesis derived from the network theory on mental disorders, associations of emotional symptoms and of emotional and cancer-treatment-related symptoms were not stronger in patients with maladaptive emotions, compared to patients with adaptive emotions.

## Supporting information

S1 Fig Contemporaneous differences hypothesis A. (DOCX)

S2 Fig Temporal differences hypothesis A. (DOCX)

S3 Fig Contemporaneous differences hypothesis B. (DOCX)

S4 Fig Temporal differences hypothesis B. (DOCX)

S5 Fig Contemporaneous differences second measurement. (DOCX)

S6 Fig Temporal differences second measurement. (DOCX)

S7 Fig Contemporaneous differences non-metastatic and metastatic disease. (DOCX)

S8 Fig Temporal differences non-metastatic and metastatic disease. (DOCX)

S9 Fig Contemporaneous differences positive emotions. (DOCX)

S10 Fig Temporal differences hypothesis positive emotions. (DOCX)

S1 Table Differences in symptom means between patients with adaptive and maladaptive emotions. (DOCX)

S2 Table Differences in symptom means between patients with non-metastatic and metastatic disease. (DOCX)

S1 Appendix Screening. (DOCX)

S2 Appendix Full EMA protocol. (DOCX)

S3 Appendix Data preparations and assumptions. (DOCX)

S4 Appendix Cross-validation. (XLSX)

S5 Appendix One-level networks. (DOCX)

S1 Matrices Matrices of the partial correlations between the negative emotions of the networks of patients with adaptive and maladaptive emotions and the differences between these partial correlations. (XLSX)

S2 Matrices Matrices of the partial correlations between the negative emotions and cancer-treatment-related symptoms of the networks of patients with adaptive and maladaptive emotions and the differences between these partial correlations. (XLSX)

S3 Matrices Matrices of the partial correlations between the positive emotions of the networks of patients with adaptive and maladaptive emotions and the differences between these partial correlations. (XLSX)

## References

[1] National Comprehensive Cancer Network. Guideline Distress Management Version 1.2024. 2023.

[2] Zabora J, BrintzenhofeSzoc K, Curbow B, Hooker C, Piantadosi S. The prevalence of psychological distress by cancer site. Psychooncology. 2001;10(1):19–28. doi: 10.1002/1099-1611(200101/02)10:1<19::aid-pon501>3.0.co;2-6 11180574

[3] Mehnert A, Hartung TJ, Friedrich M, Vehling S, Brähler E, Härter M, et al. One in two cancer patients is significantly distressed: Prevalence and indicators of distress. Psychooncology. 2018;27(1):75–82. doi: 10.1002/pon.4464 28568377

[4] Dekker J, Doppenberg-Smit E, Braamse A, Lamers F, van Linde M, Verheul HMW, et al. Toward an improved conceptualization of emotions in patients with cancer. Front Psychiatry. 2024;15:1352026. doi: 10.3389/fpsyt.2024.1352026 38600981 PMC11004313

[5] Dekker J, Braamse A, Schuurhuizen C, Beekman ATF, van Linde M, Sprangers MAG, et al. Distress in patients with cancer - on the need to distinguish between adaptive and maladaptive emotional responses. Acta Oncol. 2017;56(7):1026–9. doi: 10.1080/0284186X.2017.1280848 28145789

[6] Borsboom D. A network theory of mental disorders. World Psychiatry. 2017;16(1):5–13. doi: 10.1002/wps.20375 28127906 PMC5269502

[7] Contreras A, Nieto I, Valiente C, Espinosa R, Vazquez C. The Study of Psychopathology from the Network Analysis Perspective: A Systematic Review. Psychother Psychosom. 2019;88(2):71–83. 10.1159/000497425 30889609

[8] Robinaugh DJ, Hoekstra RHA, Toner ER, Borsboom D. The network approach to psychopathology: a review of the literature 2008-2018 and an agenda for future research. Psychol Med. 2020;50(3):353–66. doi: 10.1017/S0033291719003404 31875792 PMC7334828

[9] Neijenhuijs KI, Peeters CFW, van Weert H, Cuijpers P, Leeuw IV. Symptom clusters among cancer survivors: what can machine learning techniques tell us?. BMC Med Res Methodol. 2021;21(1):166. doi: 10.1186/s12874-021-01352-4 34399698 PMC8369803

[10] Cai T, Zhou T, Huang Q, Wu F, Ni F, Yuan C. Cancer-related symptoms among young and middle-aged women undergoing chemotherapy for breast cancer: Application of latent class analysis and network analysis. Eur J Oncol Nurs. 2023;63:102287. doi: 10.1016/j.ejon.2023.102287 36889245

[11] Bergsneider BH, Vera E, Gal O, Christ A, King AL, Acquaye A, et al. Discovery of clinical and demographic determinants of symptom burden in primary brain tumor patients using network analysis and unsupervised clustering. Neurooncol Adv. 2022;5(1):vdac188. doi: 10.1093/noajnl/vdac188 36820236 PMC9938652

[12] Doppenberg-Smit GE, van Linde ME, Lamers F, Hoogendoorn AW, Braamse AMJ, Kuip EJM, et al. Indicators of maladaptive emotions in patients with cancer as assessed by oncologists and nurses. Support Care Cancer. 2025;33(11):930. doi: 10.1007/s00520-025-09917-5 41073816 PMC12513930

[13] US Department of Health and Human Services N I o H, National Cancer Institute. Common Terminology Criteria for Adverse Events (CTCAE) Version 5. 2017.

[14] Sytema S, van der Krieke L. Routine outcome monitoring. Improving Mental Health Care. Wiley. 2013. 246–63. 10.1002/9781118337981.ch16

[15] Janssens KAM, Bos EH, Rosmalen JGM, Wichers MC, Riese H. A qualitative approach to guide choices for designing a diary study. BMC Med Res Methodol. 2018;18(1):140. doi: 10.1186/s12874-018-0579-6 30445926 PMC6240196

[16] Schoevers RA, van Borkulo CD, Lamers F, Servaas MN, Bastiaansen JA, Beekman ATF, et al. Affect fluctuations examined with ecological momentary assessment in patients with current or remitted depression and anxiety disorders. Psychol Med. 2021;51(11):1906–15. doi: 10.1017/S0033291720000689 32234092 PMC8381239

[17] Wichers M, Peeters F, Rutten BPF, Jacobs N, Derom C, Thiery E, et al. A time-lagged momentary assessment study on daily life physical activity and affect. Health Psychol. 2012;31(2):135–44. doi: 10.1037/a0025688 21988094

[18] Mehl MR, Conner TS. Handbook of research methods for studying daily life. New York: The Guilford Press. 2011.

[19] Booij SH. The Mood and Movement in Daily Life (MOOVD) Study. Groningen: University of Groningen. 2015.

[20] Bennik E. Every dark cloud has a colored lining. The relation between positive and negative affect and reactivity to positive and negative events. Groningen: University of Groningen. 2015.

[21] Item library. https://itemlibrary.eortc.org/ 2018. Accessed 2023 October 1.

[22] Wittchen HU. Reliability and validity studies of the WHO--Composite International Diagnostic Interview (CIDI): a critical review. J Psychiatr Res. 1994;28(1):57–84. doi: 10.1016/0022-3956(94)90036-1 8064641

[23] Kleinstäuber M, Exner A, Lambert MJ, Terluin B. Validation of the Four-Dimensional Symptom Questionnaire (4DSQ) in a mental health setting. Psychol Health Med. 2021;26(sup1):1–19. doi: 10.1080/13548506.2021.1883685 33835880

[24] Tuinman MA, Gazendam-Donofrio SM, Hoekstra-Weebers JE. Screening and referral for psychosocial distress in oncologic practice: use of the Distress Thermometer. Cancer. 2008;113(4):870–8. doi: 10.1002/cncr.23622 18618581

[25] van Linde ME, Braamse AMJ, Collette EH, Hoogendoorn AW, Snoek FJ, Verheul HMW, et al. Clinical assessment of emotions in patients with cancer: Diagnostic accuracy compared with two reference standards. Psychooncology. 2020;29(4):775–80. doi: 10.1002/pon.5347 32011016 PMC7216973

[26] Braamse AMJ, van Meijel B, Visser O, Huijgens PC, Beekman ATF, Dekker J. Distress, problems and supportive care needs of patients treated with auto- or allo-SCT. Bone Marrow Transplant. 2014;49(2):292–8. doi: 10.1038/bmt.2013.155 24096825

[27] R Core Team. R: A Language and Environment for Statistical Computing. https://www.R-project.org/ 2021.

[28] Hamaker EL, Grasman RPPP. Regime Switching State-Space Models Applied to Psychological Processes: Handling Missing Data and Making Inferences. Psychometrika. 2012;77(2):400–22.

[29] Epskamp S, Waldorp LJ, Mõttus R, Borsboom D. The Gaussian Graphical Model in Cross-Sectional and Time-Series Data. Multivariate Behav Res. 2018;53(4):453–80. doi: 10.1080/00273171.2018.1454823 29658809

[30] Slipetz LR, Falk A, Henry TR. Missing Data in Discrete Time State-Space Modeling of Ecological Momentary Assessment Data: A Monte-Carlo Study of Imputation Methods. Multivariate Behav Res. 2025;60(4):695–710. doi: 10.1080/00273171.2025.2469055 40091737

[31] Moritz S, Bartz-Beielstein T. ImputeTS: Time series missing value imputation in R. The R Journal. 2017;9(1):207–18.

[32] Epskamp SD, M K, Bringmann LF. Package ‘mlVAR’. https://cran.r-project.org/web/packages/mlVAR/mlVAR.pdf 2019.

[33] Epskamp S, van Borkulo CD, van der Veen DC, Servaas MN, Isvoranu A-M, Riese H, et al. Personalized Network Modeling in Psychopathology: The Importance of Contemporaneous and Temporal Connections. Clin Psychol Sci. 2018;6(3):416–27. doi: 10.1177/2167702617744325 29805918 PMC5952299

[34] Schellekens MPJ, Wolvers MDJ, Schroevers MJ, Bootsma TI, Cramer AOJ, van der Lee ML. Exploring the interconnectedness of fatigue, depression, anxiety and potential risk and protective factors in cancer patients: a network approach. J Behav Med. 2020;43(4):553–63. doi: 10.1007/s10865-019-00084-7 31435892 PMC7366596

[35] Haslbeck JMB. Mnet: Modelling Group Differences and Moderation Effects in Statistical Network Models. https://cran.r-project.org/web/packages/mnet/index.html 2023.

[36] Haslbeck J, Epskamp S, Waldorp L. Testing for group differences in multilevel vector autoregressive models. 2023. 10.3758/s13428-024-02541-x 39979514

[37] van Borkulo CD, van Bork R, Boschloo L, Kossakowski JJ, Tio P, Schoevers RA, et al. Comparing network structures on three aspects: A permutation test. Psychol Methods. 2023;28(6):1273–85. doi: 10.1037/met0000476 35404628

[38] Shiffman S, Stone AA, Hufford MR. Ecological momentary assessment. Annu Rev Clin Psychol. 2008;4:1–32. doi: 10.1146/annurev.clinpsy.3.022806.091415 18509902

[39] Epskamp S, Borsboom D, Fried EI. Estimating psychological networks and their accuracy: A tutorial paper. Behav Res Methods. 2018;50(1):195–212. doi: 10.3758/s13428-017-0862-1 28342071 PMC5809547

[40] Scott AJ, Correa AB, Bisby MA, Dear BF. Depression and Anxiety Trajectories in Chronic Disease: A Systematic Review and Meta-Analysis. Psychother Psychosom. 2023;92(4):227–42. doi: 10.1159/000533263 37607505

[41] Herschbach P, Britzelmeir I, Dinkel A, Giesler JM, Herkommer K, Nest A, et al. Distress in cancer patients: Who are the main groups at risk?. Psychooncology. 2020;29(4):703–10. doi: 10.1002/pon.5321 31876011

[42] Lotfi-Jam K, Gough K, Schofield P, Aranda S, Jefford M. A longitudinal study of four unique trajectories of psychological distress in cancer survivors after completing potentially curative treatment. Acta Oncol. 2019;58(5):782–9. doi: 10.1080/0284186X.2018.1562209 30698063

[43] Bringmann LF, Albers C, Bockting C, Borsboom D, Ceulemans E, Cramer A, et al. Psychopathological networks: Theory, methods and practice. Behav Res Ther. 2022;149:104011. doi: 10.1016/j.brat.2021.104011 34998034

[44] Jones PJ, Heeren A, McNally RJ. Commentary: A network theory of mental disorders. Front Psychol. 2017;8:1305. doi: 10.3389/fpsyg.2017.01305 28824490 PMC5539126

[45] Wichers M, Riese H, Hodges TM, Snippe E, Bos FM. A Narrative Review of Network Studies in Depression: What Different Methodological Approaches Tell Us About Depression. Front Psychiatry. 2021;12:719490. doi: 10.3389/fpsyt.2021.719490 34777038 PMC8581034

[46] Doppenberg-Smit GE, Lamers F, van Linde ME, Braamse AMJ, Sprangers MAG, Beekman ATF, et al. Network analysis used to investigate the interplay among somatic and psychological symptoms in patients with cancer and cancer survivors: a scoping review. J Cancer Surviv. 2024;19(4):1198–211. doi: 10.1007/s11764-024-01543-0 PMC12283480 38530627

