# Supplementary material for: Adaptive and maladaptive emotions in patients with cancer: A network perspective
Source: PLoS One. 2026 Oct 8;21(10):e0341290. doi: 10.1371/journal.pone.0341290 (PMC13649014; doi:10.1371/journal.pone.0341290)
Supplement: Supplementary file 6 [file pone.0341290.s006.docx]

Supplemental Figure 6. Differences in temporal networks including negative emotional and cancer-treatment-related symptoms for the second measurement.

Figure S6a. Significant differences between adaptive and maladaptive temporal networks (second measurement) based on the psychiatric diagnostic assessment reference standard. Red lines indicate a stronger edge in the maladaptive network, green lines indicate stronger edges in the adaptive network. Negative emotional symptoms: "Anx" = "Anxious", "Brd" = "Bored", "Dwn" = "Down","Irr" = "Irritated","Lst" = "Listless", "Nrv" = "Nervous", "Ups" = "Upset", "Wrr" = "Worried"; cancer-treatment-related symptoms: "LCc" = "Lack of concentration", "Nss" = "Nauseous", "LcA" = "Lack of appetite", "Pan" = "Pain", "ShB" = "Shortness of breath", "Trd" = "Tired".

Figure S6b. Significant differences between adaptive and maladaptive temporal networks (second measurement) based on the subjective need for professional mental healthcare reference standard. Red lines indicate a stronger edge in the maladaptive network, green lines indicate stronger edges in the adaptive network. Negative emotional symptoms: "Anx" = "Anxious", "Brd" = "Bored", "Dwn" = "Down","Irr" = "Irritated","Lst" = "Listless", "Nrv" = "Nervous", "Ups" = "Upset", "Wrr" = "Worried"; cancer-treatment-related symptoms: "LCc" = "Lack of concentration", "Nss" = "Nauseous", "LcA" = "Lack of appetite", "Pan" = "Pain", "ShB" = "Shortness of breath", "Trd" = "Tired".
