# Supplementary material for: Adaptive and maladaptive emotions in patients with cancer: A network perspective
Source: PLoS One. 2026 Oct 8;21(10):e0341290. doi: 10.1371/journal.pone.0341290 (PMC13649014; doi:10.1371/journal.pone.0341290)
Supplement: Supplementary file 9 [file pone.0341290.s009.docx]

Supplemental Figure 9. Differences in contemporaneous networks including positive emotions.

Figure S9a. Significant differences between adaptive and maladaptive cotemporaneous networks (Figure 6) based on the psychiatric diagnostic assessment reference standard. Red lines indicate a stronger edge in the maladaptive network, green lines indicate stronger edges in the adaptive network. "Chr" = "Cheerful", "Clm" = "Calm", "Enr" = "Energetic", "Ent" = "Enthusiastic", "Rlx" = "Relaxed","Sts" = "Satisfied".

Figure S9b. Significant differences between adaptive and maladaptive contemporaneous networks (Figure 6) based on the subjective need for professional mental healthcare reference standard. Red lines indicate a stronger edge in the maladaptive network, green lines indicate stronger edges in the adaptive network. "Chr" = "Cheerful", "Clm" = "Calm", "Enr" = "Energetic", "Ent" = "Enthusiastic", "Rlx" = "Relaxed","Sts" = "Satisfied".
