# Supplementary material for: Adaptive and maladaptive emotions in patients with cancer: A network perspective
Source: PLoS One. 2026 Oct 8;21(10):e0341290. doi: 10.1371/journal.pone.0341290 (PMC13649014; doi:10.1371/journal.pone.0341290)
Supplement: Supplementary file 10 [file pone.0341290.s010.docx]

Supplemental Figure 10. Differences in temporal networks including positive emotions.

Figure S10a. Significant differences between adaptive (Figure 6a) and maladaptive temporal networks (Figure 7) based on the psychiatric diagnostic assessment reference standard. Red lines indicate a stronger edge in the maladaptive network, green lines indicate stronger edges in the adaptive network. "Chr" = "Cheerful", "Clm" = "Calm", "Enr" = "Energetic", "Ent" = "Enthusiastic", "Rlx" = "Relaxed","Sts" = "Satisfied".

Figure S10b. Significant differences between adaptive and maladaptive temporal networks (Figure 7) based on the subjective need for professional mental healthcare reference standard. Red lines indicate a stronger edge in the maladaptive network, green lines indicate stronger edges in the adaptive network. "Chr" = "Cheerful", "Clm" = "Calm", "Enr" = "Energetic", "Ent" = "Enthusiastic", "Rlx" = "Relaxed","Sts" = "Satisfied".
