# Supplementary material for: Adaptive and maladaptive emotions in patients with cancer: A network perspective
Source: PLoS One. 2026 Oct 8;21(10):e0341290. doi: 10.1371/journal.pone.0341290 (PMC13649014; doi:10.1371/journal.pone.0341290)
Supplement: Supplementary file 11 [file pone.0341290.s011.docx]

| Symptom | Group | Mean | SD | Mann Whitney U test, p-value |
| --- | --- | --- | --- | --- |
| Anxious | Adaptive emotions | 1.33 | 0.63 | W = 951.5, p < 0.001 |
|  | Maladaptive emotions | 1.85 | 0.91 |  |
|  | Total | 1.45 | 0.74 |  |
| Bored | Adaptive emotions | 1.29 | 0.43 | W = 1026, p = 0.003 |
|  | Maladaptive emotions | 1.83 | 0.93 |  |
|  | Total | 1.42 | 0.62 |  |
| Down | Adaptive emotions | 1.54 | 0.70 | W = 665.5, p < 0.001 |
|  | Maladaptive emotions | 2.54 | 1.15 |  |
|  | Total | 1.77 | 0.93 |  |
| Irritated | Adaptive emotions | 1.37 | 0.54 | W = 984, p = 0.002 |
|  | Maladaptive emotions | 1.81 | 0.84 |  |
|  | Total | 1.47 | 0.65 |  |
| Listless | Adaptive emotions | 1.99 | 0.97 | W = 1041, p = 0.005 |
|  | Maladaptive emotions | 2.63 | 1.23 |  |
|  | Total | 2.14 | 1.07 |  |
| Nervous | Adaptive emotions | 1.43 | 0.65 | W = 8.37, p < 0.001 |
|  | Maladaptive emotions | 2.00 | 0.94 |  |
|  | Total | 1.57 | 0.76 |  |
| Upset | Adaptive emotions | 1.25 | 0.44 | W = 981.5, p = 0.002 |
|  | Maladaptive emotions | 1.68 | 0.78 |  |
|  | Total | 1.35 | 0.57 |  |
| Worried | Adaptive emotions | 1.99 | 1.07 | W = 803, p < 0.001 |
|  | Maladaptive emotions | 3.04 | 1.39 |  |
|  | Total | 2.24 | 1.23 |  |

(a) Psychiatric diagnostic assessment negative emotions

Note. N^Adaptive^ = 101, N^Maladaptive^ = 31, N^Total^ = 132

(b) Subjective need for professional mental healthcare negative emotions

| Symptom | Group | Mean | SD | Mann Whitney U test, p-value |
| --- | --- | --- | --- | --- |
| Anxious | Adaptive emotions | 1.33 | 0.60 | W = 1071.5, p < 0.001 |
|  | Maladaptive emotions | 1.78 | 0.96 |  |
|  | Total | 1.45 | 0.74 |  |
| Bored | Adaptive emotions | 1.38 | 0.60 | W = 1404.5, p = 0.128 |
|  | Maladaptive emotions | 1.54 | 0.68 |  |
|  | Total | 1.42 | 0.62 |  |
| Down | Adaptive emotions | 1.61 | 0.79 | W = 1106.5, p = 0.002 |
|  | Maladaptive emotions | 2.23 | 1.12 |  |
|  | Total | 1.77 | 0.93 |  |
| Irritated | Adaptive emotions | 1.39 | 0.55 | W = 1309.5, p = 0.045 |
|  | Maladaptive emotions | 1.69 | 0.83 |  |
|  | Total | 1.47 | 0.65 |  |
| Listless | Adaptive emotions | 2.08 | 1.04 | W = 1450.5, p = 0.204 |
|  | Maladaptive emotions | 2.31 | 1.15 |  |
|  | Total | 2.14 | 1.07 |  |
| Nervous | Adaptive emotions | 1.47 | 0.67 | W = 1225.5, p = 0.015 |
|  | Maladaptive emotions | 1.83 | 0.93 |  |
|  | Total | 1.57 | 0.76 |  |
| Upset | Adaptive emotions | 1.28 | 0.49 | W = 113, p = 0.002 |
|  | Maladaptive emotions | 1.55 | 0.71 |  |
|  | Total | 1.35 | 0.57 |  |
| Worried | Adaptive emotions | 2.12 | 1.22 | W = 1290, p = 0.036 |
|  | Maladaptive emotions | 2.54 | 1.22 |  |
|  | Total | 2.24 | 1.23 |  |

Note. N^Adaptive^ = 97, N^Maladaptive^ = 35, N^Total^ = 132

(c) Psychiatric diagnostic assessment cancer-treatment-related symptoms

| Symptom | Group | Mean | SD | Mann Whitney U test, p-value |
| --- | --- | --- | --- | --- |
| Lack of Appetite | Adaptive emotions | 1.63 | 1.06 | W = 1308.5, p = 0.160 |
|  | Maladaptive emotions | 2.04 | 1.45 |  |
|  | Total | 1.73 | 1.17 |  |
| Lack of Concentration | Adaptive emotions | 2.23 | 1.34 | W = 888, p <0.001 |
|  | Maladaptive emotions | 3.27 | 1.45 |  |
|  | Total | 2.47 | 1.43 |  |
| Nauseous | Adaptive emotions | 1.33 | 0.72 | W = 1226, p = 0.060 |
|  | Maladaptive emotions | 1.49 | 0.75 |  |
|  | Total | 1.36 | 0.73 |  |
| Pain | Adaptive emotions | 1.89 | 1.25 | W = 1217.5, p = 0.061 |
|  | Maladaptive emotions | 2.20 | 1.22 |  |
|  | Total | 1.96 | 1.24 |  |
| Shortness  of Breath | Adaptive emotions | 1.72 | 1.23 | W = 1379, p = 0.300 |
|  | Maladaptive emotions | 1.90 | 1.29 |  |
|  | Total | 1.76 | 1.24 |  |
| Tired | Adaptive emotions | 3.51 | 1.37 | W = 1268.5, p = 0.112 |
|  | Maladaptive emotions | 3.95 | 1.33 |  |
|  | Total | 3.62 | 1.37 |  |

Note. N^Adaptive^ = 101, N^Maladaptive^ = 31, N^Total^ = 132

(d) Subjective need for professional mental healthcare cancer-treatment-related symptoms

| Symptom | Group | Mean | SD | Mann Whitney U test, p-value |
| --- | --- | --- | --- | --- |
| Lack of Appetite | Adaptive emotions | 1.69 | 1.11 | W = 1585, p = 0.556 |
|  | Maladaptive emotions | 1.83 | 1.12 |  |
|  | Total | 1.73 | 1.17 |  |
| Lack of Concentration | Adaptive emotions | 2.36 | 1.35 | W = 1343.5, p = 0.068 |
|  | Maladaptive emotions | 2.80 | 1.60 |  |
|  | Total | 2.47 | 1.43 |  |
| Nauseous | Adaptive emotions | 1.35 | 0.67 | W = 1590.5, p = 0.570 |
|  | Maladaptive emotions | 1.40 | 0.88 |  |
|  | Total | 1.36 | 0.73 |  |
| Pain | Adaptive emotions | 2.00 | 1.49 | W = 1869.5, p = 0.374 |
|  | Maladaptive emotions | 1.86 | 1.26 |  |
|  | Total | 1.36 | 0.73 |  |
| Shortness  of Breath | Adaptive emotions | 1.73 | 1.19 | W = 1827.5, p = 0.486 |
|  | Maladaptive emotions | 1.84 | 1.37 |  |
|  | Total | 1.76 | 1.24 |  |
| Tired | Adaptive emotions | 3.54 | 1.43 | W = 1520, p = 0.362 |
|  | Maladaptive emotions | 3.83 | 1.18 |  |
|  | Total | 3.62 | 1.37 |  |

Note. N^Adaptive^ = 97, N^Maladaptive^ = 35, N^Total^ = 132

(e) Psychiatric diagnostic assessment positive emotional symptoms

| Symptom | Group | Mean | SD | Mann Whitney U test, p-value |  |
| --- | --- | --- | --- | --- | --- |
| Calm | Adaptive emotions | 4.98 | 0.78 | W = 2287, p < 0.001 |  |
|  | Maladaptive emotions | 4.28 | 0.97 |  |  |
|  | Total | 4.81 | 0.88 |  |  |
| Cheerful | Adaptive emotions | 4.79 | 0.93 | W = 2349.5, p < 0.001 |  |
|  | Maladaptive emotions | 3.87 | 1.12 |  |  |
|  | Total | 4.57 | 1.05 |  |  |
| Energetic | Adaptive emotions | 4.35 | 0.98 | W = 2242.5, p <0.001 |  |
|  | Maladaptive emotions | 3.52 | 1.14 |  |  |
|  | Total | 4.16 | 1.07 |  |  |
| Enthusiastic | Adaptive emotions | 4.48 | 1.00 | w = 2282.5, p < 0.001 |  |
|  | Maladaptive emotions | 3.55 | 1.18 |  |  |
|  | Total | 4.26 | 1.12 |  |  |
| Relaxed | Adaptive emotions | 5.29 | 0.70 | W = 2316, p < 0.001 |  |
|  | Maladaptive emotions | 4.56 | 0.88 |  |  |
|  | Total | 5.11 | 0.80 |  |  |
|  | Adaptive emotions | 5.39 | 0.70 | W = 2321.5, p <0.001 |  |
| Satisfied | Maladaptive emotions | 4.66 | 0.91 |  |  |
|  | Total | 5.22 | 0.81 |  |  |

Note. N^Adaptive^ = 101, N^Maladaptive^ = 31, N^Total^ = 132

(f) Subjective need for professional mental healthcare positive emotional symptoms

| Symptom | Group | Mean | SD | Mann Whitney U test, p-value |
| --- | --- | --- | --- | --- |
| Calm | Adaptive emotions | 4.89 | 0.87 | W = 2073.5, p = 0.053 |
|  | Maladaptive emotions | 4.59 | 0.87 |  |
|  | Total | 4.81 | 0.88 |  |
| Cheerful | Adaptive emotions | 4.72 | 0.91 | W = 2113, p = 0.032 |
|  | Maladaptive emotions | 4.17 | 1.29 |  |
|  | Total | 4.57 | 1.05 |  |
| Energetic | Adaptive emotions | 4.24 | 1.02 | W = 1961.5, p = 0.174 |
|  | Maladaptive emotions | 3.92 | 1.19 |  |
|  | Total | 4.16 | 1.07 |  |
| Enthusiastic | Adaptive emotions | 4.37 | 1.03 | W = 1980, p = 0.146 |
|  | Maladaptive emotions | 3.98 | 1.29 |  |
|  | Total | 4.26 | 1.12 |  |
| Relaxed | Adaptive emotions | 5.19 | 0.74 | W = 1982, p = 0.143 |
|  | Maladaptive emotions | 4.91 | 0.94 |  |
|  | Total | 5.11 | 0.80 |  |
|  | Adaptive emotions | 5.31 | 0.73 | W = 2044.5, p = 0.074 |
| Satisfied | Maladaptive emotions | 4.96 | 0.98 |  |
|  | Total | 5.22 | 0.81 |  |

Note. N^Adaptive^ = 97, N^Maladaptive^ = 35, N^Total^ = 132
