# Supplementary material for: Adaptive and maladaptive emotions in patients with cancer: A network perspective
Source: PLoS One. 2026 Oct 8;21(10):e0341290. doi: 10.1371/journal.pone.0341290 (PMC13649014; doi:10.1371/journal.pone.0341290)
Supplement: Supplementary file 12 [file pone.0341290.s012.docx]

| Symptom | Group | Mean | SD | T-test, p-value |
| --- | --- | --- | --- | --- |
| Anxious | Non-metastatic disease | 1.46 | 0.75 | t(130) = 0.12, p = 0.907 |
|  | Metastatic disease | 1.44 | 0.73 |  |
|  | Total | 1.45 | 0.74 |  |
| Bored | Non-metastatic disease | 1.43 | 0.58 | t(130) = 0.19, p = 0.849 |
|  | Metastatic disease | 1.41 | 0.69 |  |
|  | Total | 1.42 | 0.62 |  |
| Down | Non-metastatic disease | 1.79 | 0.93 | t(130) = 0.25, p = 0.804 |
|  | Metastatic disease | 1.75 | 0.92 |  |
|  | Total | 1.77 | 0.93 |  |
| Irritated | Non-metastatic disease | 1.53 | 0.67 | t(130) = 1.13, p = 0.262 |
|  | Metastatic disease | 1.40 | 0.60 |  |
|  | Total | 1.47 | 0.65 |  |
| Listless | Non-metastatic disease | 2.19 | 1.05 | t(130) = 0.64, p = 0.525 |
|  | Metastatic disease | 2.07 | 1.10 |  |
|  | Total | 2.14 | 1.07 |  |
| Nervous | Non-metastatic disease | 1.62 | 0.82 | t(130) = 0.98, p = 0.327 |
|  | Metastatic disease | 1.49 | 0.67 |  |
|  | Total | 1.57 | 0.76 |  |
| Upset | Non-metastatic disease | 1.37 | 0.56 | t(130) = 0.34, p – 0.736 |
|  | Metastatic disease | 1.33 | 0.59 |  |
|  | Total | 1.35 | 0.57 |  |
| Worried | Non-metastatic disease | 2.30 | 1.33 | t(130) = 0.73, p = 0.468 |
|  | Metastatic disease | 2.15 | 1.10 |  |
|  | Total | 2.24 | 1.23 |  |

(a) Negative emotional symptoms

Note. N^non-metastastic^ = 74, N^Metastatic^ = 58, N^Total^ = 132

| Symptom | Group | Mean | SD | T-test, p-value |
| --- | --- | --- | --- | --- |
| Lack of Appetite | Non-metastatic disease | 1.87 | 1.29 | t(130) = 1.62, p = 0.107 |
|  | Metastatic disease | 1.54 | 0.97 |  |
|  | Total | 1.73 | 1.17 |  |
| Lack of Concentration | Non-metastatic disease | 2.67 | 1.44 | t(130) = 1.84, p = 0.068 |
|  | Metastatic disease | 2.22 | 1.39 |  |
|  | Total | 2.47 | 1.43 |  |
| Nauseous | Non-metastatic disease | 1.38 | 0.68 | t(130) = 0.22, p = 0.823 |
|  | Metastatic disease | 1.35 | 0.79 |  |
|  | Total | 1.36 | 0.73 |  |
| Pain | Non-metastatic disease | 1.93 | 1.27 | t(130) = -0.34, p = 0.737 |
|  | Metastatic disease | 2.00 | 1.22 |  |
|  | Total | 1.96 | 1.24 |  |
| Shortness  of Breath | Non-metastatic disease | 1.52 | 0.95 | t(130) = -2.59, p = 0.011 |
|  | Metastatic disease | 2.07 | 1.48 |  |
|  | Total | 1.76 | 1.24 |  |
| Tired | Non-metastatic disease | 3.72 | 1.30 | t(130) = 0.99, p = 0.324 |
|  | Metastatic disease | 3.48 | 1.45 |  |
|  | Total | 3.62 | 1.37 |  |

(b) Cancer-treatment-related symptoms

Note. N^non-metastastic^ = 74, N^Metastatic^ = 58, N^Total^ = 132

(c) Positive emotional symptoms

| Symptom | Group | Mean | SD | T-test, p-value |
| --- | --- | --- | --- | --- |
| Calm | Non-metastatic disease | 4.78 | 0.98 | t(130) = -0.55, p = 0.584 |
|  | Metastatic disease | 4.86 | 0.73 |  |
|  | Total | 4.81 | 0.88 |  |
| Cheerful | Non-metastatic disease | 4.37 | 1.14 | t(130) = -2.51, p = 0.012 |
|  | Metastatic disease | 4.83 | 0.88 |  |
|  | Total | 4.57 | 1.05 |  |
| Energetic | Non-metastatic disease | 3.99 | 1.05 | t(130) = -2.04, p = 0.044 |
|  | Metastatic disease | 4.37 | 1.07 |  |
|  | Total | 4.16 | 1.07 |  |
| Enthusiastic | Non-metastatic disease | 4.07 | 1.15 | t(130) = -2.30, p = 0.023 |
|  | Metastatic disease | 4.51 | 1.02 |  |
|  | Total | 4.26 | 1.12 |  |
| Relaxed | Non-metastatic disease | 5.06 | 0.84 | t(130) = -0.84, p = 0.401 |
|  | Metastatic disease | 5.18 | 0.76 |  |
|  | Total | 5.11 | 0.80 |  |
| Satisfied | Non-metastatic disease | 5.16 | 0.82 | t(130) = -0.90, p = 0.370 |
|  | Metastatic disease | 5.29 | 0.80 |  |
|  | Total | 5.22 | 0.81 |  |

Note. N^non-metastastic^ = 74, N^Metastatic^ = 58, N^Total^ = 132
