# Supplementary material for: Adaptive and maladaptive emotions in patients with cancer: A network perspective
Source: PLoS One. 2026 Oct 8;21(10):e0341290. doi: 10.1371/journal.pone.0341290 (PMC13649014; doi:10.1371/journal.pone.0341290)
Supplement: Supplementary file 13 [file pone.0341290.s013.docx]

**S1 Appendix. Screening.**

We aimed to include 80 patients with adaptive and 80 with maladaptive emotions. Because patients with maladaptive emotions are a minority, after 80% of patients with adaptive emotions were included we screened for patients with maladaptive emotions using the extended version of the Kessler-10 (EK-10) (1) and with the subjective need for professional mental healthcare question. Patients could only be included from that point onwards if they were screen-positive on the EK-10 (a score ≥ 20, or a positive answer on one of the five additional questions indicating distress (1)), or if they indicated to currently had or wished to have a referral because of their emotional symptoms (as part of the subjective need for professional mental healthcare questions).

The Kessler-10 is a validated screening to screen for emotional distress. It is likely that patients who screen positive experience maladaptive emotions. This is however not a guarantee. Therefore, we expected to fill the remainder 20 % of the adaptive group with patients who are screen-positive, but turn out to fall within the adaptive group upon participation.

Because we also include patients’ subjective need for professional mental healthcare, we included an additional question directly asking whether they had a referral of wished to have a referral for their emotional problems.

Reference:

1.	Donker T, Comijs H, Cuijpers P, Terluin B, Nolen W, Zitman F, et al. The validity of the Dutch K10 and extended K10 screening scales for depressive and anxiety disorders. Psychiatry Res. 2010;176(1):45-50.
