# Supplementary material for: Adaptive and maladaptive emotions in patients with cancer: A network perspective
Source: PLoS One. 2026 Oct 8;21(10):e0341290. doi: 10.1371/journal.pone.0341290 (PMC13649014; doi:10.1371/journal.pone.0341290)
Supplement: Supplementary file 14 [file pone.0341290.s014.docx]

*S2 Appendix. Full EMA protocol*

*The first part of this questionnaire is about how you feel right now.*

**Q1 I feel satisfied:**
1	2	3	4	5	6	7
⃝	⃝	⃝	⃝	⃝	⃝	⃝
not at all	moderate	very much

**Q2 I feel relaxed:**
1	2	3	4	5	6	7
⃝	⃝	⃝	⃝	⃝	⃝	⃝
not at all	moderate	very much

**Q3 I feel upset:**
1	2	3	4	5	6	7
⃝	⃝	⃝	⃝	⃝	⃝	⃝
not at all	moderate	very much

**Q4 I feel cheerful:**
1	2	3	4	5	6	7
⃝	⃝	⃝	⃝	⃝	⃝	⃝
not at all	moderate	very much

**Q5 I feel irritated:**
1	2	3	4	5	6	7
⃝	⃝	⃝	⃝	⃝	⃝	⃝
not at all	moderate	very much

**Q6 I feel listless:**
1	2	3	4	5	6	7
⃝	⃝	⃝	⃝	⃝	⃝	⃝
not at all	moderate	very much

**Q7 I feel down:**
1	2	3	4	5	6	7
⃝	⃝	⃝	⃝	⃝	⃝	⃝
not at all	moderate	very much

**Q8 I feel energetic:**
1	2	3	4	5	6	7
⃝	⃝	⃝	⃝	⃝	⃝	⃝
not at all	moderate	very much
 **Q9 I feel enthusiastic:**
1	2	3	4	5	6	7
⃝	⃝	⃝	⃝	⃝	⃝	⃝
not at all	moderate	very much

**Q10 I feel nervous:**
1	2	3	4	5	6	7
⃝	⃝	⃝	⃝	⃝	⃝	⃝
not at all	moderate	very much

**Q11 I feel bored:**
1	2	3	4	5	6	7
⃝	⃝	⃝	⃝	⃝	⃝	⃝
not at all	moderate	very much

**Q12 I feel calm:**
1	2	3	4	5	6	7
⃝	⃝	⃝	⃝	⃝	⃝	⃝
not at all	moderate	very much

**Q13 I feel anxious:**
1	2	3	4	5	6	7
⃝	⃝	⃝	⃝	⃝	⃝	⃝
not at all	moderate	very much

**Q14 I worry:**
1	2	3	4	5	6	7
⃝	⃝	⃝	⃝	⃝	⃝	⃝
not at all	moderate	very much

**Q15 I feel sad:**
1	2	3	4	5	6	7
⃝	⃝	⃝	⃝	⃝	⃝	⃝
not at all	moderate	very much

*The next questions are about how your physcial symptoms are right now.*

**Q16 I feel tired:**
1	2	3	4	5	6	7
⃝	⃝	⃝	⃝	⃝	⃝	⃝
not at all	moderate	very much

**Q17 I have difficulty concentrating**
1	2	3	4	5	6	7
⃝	⃝	⃝	⃝	⃝	⃝	⃝
not at all	moderate	very much

**Q18 I feel nauseous:**
1	2	3	4	5	6	7
⃝	⃝	⃝	⃝	⃝	⃝	⃝
not at all	moderate	very much

**Q19 I have a lack of appetite:**
1	2	3	4	5	6	7
⃝	⃝	⃝	⃝	⃝	⃝	⃝
not at all	moderate	very much

**Q20 I have pain:**
1	2	3	4	5	6	7
⃝	⃝	⃝	⃝	⃝	⃝	⃝
not at all	moderate	very much

**Q21 I have shortness of breath:**
1	2	3	4	5	6	7
⃝	⃝	⃝	⃝	⃝	⃝	⃝
not at all	moderate	very much

**Q22 Where are you at the moment?**
⃝ at home

⃝ work/study

⃝ at the house of people I know

⃝ public building

⃝ on the road (travel)

⃝ outside (other than traveling)

**Q23a with whom are you at the moment?**
⃝ alone

⃝ in company with others

[Follow-up question Q23b]
**Q23b The company i’m in consists of:** (multiple anwers possible)
□ partner

□ resident family members

□ family living away from home

□ friends

□ acquantances

□ strangers

□ pet(s)

**Q24 which of the following activties have you done since the last assessment? (multiple answers possible)**

□ work/study activities

□ Activties related to the treatment (example: going to the hospital)

□ Activtities related to health (example: working out/diet)

□ Social activities with family/friends

□ Intellectual/spiritual/religious activities

□ Leisure activities

□ I did not do any activities, even though I did want to

□ I did not do any acitivities

**Q25 How did you experience filling out this questionnaire?**
1	2	3	4	5	6	7
⃝	⃝	⃝	⃝	⃝	⃝	⃝
Not at all	neutral	very much enjoyable
enjoyable

**Q26 If you want you can leave comments here.**

**Thank you very much for filling out the questionnaire and do not forget to charge your phone.**
