# Supplementary material for: Adaptive and maladaptive emotions in patients with cancer: A network perspective
Source: PLoS One. 2026 Oct 8;21(10):e0341290. doi: 10.1371/journal.pone.0341290 (PMC13649014; doi:10.1371/journal.pone.0341290)
Supplement: Supplementary file 15 [file pone.0341290.s015.docx]

**Assumptions checked:**

- Stationarity

Time series are stationary when average and standard deviation are similar over time. The Augmented Dickey-Fuller (ADF) test was used to check stationarity (1).

- Variability

For sufficient variability, a rule of 10% of the scale as variance was used. Because a Likert scale from 1 to 7 was used, 0.6 was the cut off to be met.

**Cross-validation of the results**

Because not all variables showed sufficient variability, we explored the robustness of the results by cross validating the analyses for testing by doing 1000 iterations of the analysis with each time a random sample of 80% of the patients. We tested the contemporaneous and temporal networks of both reference standards (psychiatric diagnostic assessment and subjective need for professional healthcare) for the networks with emotional symptoms. For each network we checked for every possible edge how often it was present out of a possible 1000. We then compared it to the results of the generated networks. For each edge, if it was visible in the generated network we checked whether it was also present in >800 of the 1000 generated network. A percentage was calculated of how often the edges of the generated network was also present in >800 of the cross-validated networks. The results are shown in S3 Appendix.

Reference

1.	Vaisla DK, Bhatt DA. An Analysis of the Performance of Artificial Neural Network Technique for Stock Market Forecasting. International Journal on Computer Science and Engineering. 2010.
