# Supplementary material for: Adaptive and maladaptive emotions in patients with cancer: A network perspective
Source: PLoS One. 2026 Oct 8;21(10):e0341290. doi: 10.1371/journal.pone.0341290 (PMC13649014; doi:10.1371/journal.pone.0341290)
Supplement: Supplementary file 16 [file pone.0341290.s017.docx]

**Networks based on psychiatric diagnostic assessment reference standard**

Network of patients with adaptive emotions

Global strength = 3.57

Network of patients with maladaptive emotions

Global strength = 4.59

**Network Comparison Test**

Difference in global strength 1.02, p = 0.06

**Networks based on the subjective need for professional mental healthcare reference standard**

Network of patients with adaptive emotions

Global strength = 3.79

Network of patients with maladaptive emotions

Global strength = 3.66

**Network Comparison Test**

Difference in global strength = 0.13, p = 0.89
